# Interaction between Dysfunctional Connectivity at Rest and Heroin Cues-Induced Brain Responses in Male Abstinent Heroin-Dependent Individuals

**DOI:** 10.1371/journal.pone.0023098

**Published:** 2011-10-18

**Authors:** Jixin Liu, Wei Qin, Kai Yuan, Jing Li, Wei Wang, Qiang Li, Yarong Wang, Jinbo Sun, Karen M. von Deneen, Yijun Liu, Jie Tian

**Affiliations:** 1 Life Sciences Research Center, School of Life Sciences and Technology, Xidian University, Xi'an, Shaanxi, China; 2 The Fourth Military Medical University, Xi'an, Shaanxi, China; 3 Departments of Psychiatry and Neuroscience, McKnight Brain Institute, University of Florida, Gainesville, Florida, United States of America; 4 Institute of Automation, Chinese Academy of Sciences, Beijing, China; University of California San Francisco, United States of America

## Abstract

**Background:**

The majority of previous heroin cue-reactivity functional magnetic resonance imaging (fMRI) studies focused on local function impairments, such as inhibitory control, decision-making and stress regulation. Our previous studies have demonstrated that these brain circuits also presented dysfunctional connectivity during the resting state. Yet few studies considered the relevance of resting state dysfunctional connectivity to task-related neural activity in the same chronic heroin user (CHU).

**Methodology/Principal Findings:**

We employed the method of graph theory analysis, which detected the abnormality of brain regions and dysregulation of brain connections at rest between 16 male abstinent chronic heroin users (CHUs) and 16 non-drug users (NDUs). Using a cue-reactivity task, we assessed the relationship between drug-related cue-induced craving activity and the abnormal topological properties of the CHUs' resting networks. Comparing NDUs' brain activity to that of CHUs, the intensity of functional connectivity of the medial frontal gyrus (meFG) in patients' resting state networks was prominently greater and positively correlated with the same region's neural activity in the heroin-related task; decreased functional connectivity intensity of the anterior cingulate cortex (ACC) in CHUs at rest was associated with more drug-related cue-induced craving activities.

**Conclusions:**

These results may indicate that there exist two brain systems interacting simultaneously in the heroin-addicted brain with regards to a cue-reactivity task. The current study may shed further light on the neural architecture that supports craving responses in heroin dependence.

## Introduction

Heroin addiction is a complex disease of the brain, involving both affective and cognitive processes, characterized by a compulsive drive to take drugs despite serious negative consequences [1]. Emerging neuroimaging studies viewed heroin addiction under a cue-reactivity paradigm in which drug-related cues caused significant psychophysiological reactions. The impaired response inhibition function and decision-making function were found in heroin-dependent patients, which were marked by abnormal activation of the prefrontal cortex (PFC) and anterior cingulate cortex (ACC) in certain tasks [2]–[5]. Our group previously focused on resting state abnormalities in chronic heroin users (CHUs) and assessed the relationship between the resting state functional connectivity changes and duration of heroin use [5]–[8]. We also found dysregulated functional connectivity of the ACC and PFC in the CHUs' resting networks [5], [6], [8]. Based on these inherent features, however, whether or not the relevance of resting state dysfunctional connectivity is related to specific heroin cue reactivity in heroin dependent patients is still unclear.

Recent studies have suggested that resting state activity had a specific impact upon subsequent task-induced activity and may be relevant to individual variability in behavioral and mental states [9]–[14]. For example, Wang et al. (2010) demonstrated that stronger connectivity between the hippocampal and posteromedial regions during rest predicted better performance on the memory task in cognitively-intact older individuals [13]. Seeley et al. (2007) calculated the correlation between intrinsic resting functional connectivity and an individual's prescan anxiety ratings, identifying two dissociable networks in humans that are critical for guidance of thought and behavior [15]. Comprehensive investigation of brain responses from a rest-task interactions view would allow for a more general, integrated understanding of the mechanisms underlying diseased mental states [12]. While functional abnormality has been reported in resting state networks and cue-induced tasks in heroin-dependent individuals, therefore, the first aim of this study was to examine the direct relationship between abnormal resting functional connectivity and the brain response to heroin cue reactivity. Few studies have directly addressed how resting state activity interacts with stimulus-induced activity in the same CHU, and little is known about the relevance of resting state dysfunctional connectivity to task-related neural activity. We hypothesized that the changes in resting state networks would correlate with heroin cue-induced activity in CHUs.

Long-term heroin dependence impairs cortical and subcortical limbic/paralimbic brain regions involved in emotion, reward, motivation and impulse control [1], [8], [16]. Volkow et al.(2003) considered drug addiction as a state initiated by the qualitatively different and larger reward value of the drug, which triggered a series of adaptations and changes in motivation, memory, and control circuits of the brain [6], [17]. These brain circuits may also exhibit abnormal function in heroin cue processes. Therefore, our second aim was to investigate the different modes of these brain networks and their distinct interaction patterns between rest and heroin cue response processes. We hope to gain deeper insight into the neural architecture that supports fundamental aspects of human behavior in CHUs [15].

To characterize the rest-task interaction in CHUs, we used the method of graph theory analysis (GTA), which has become a powerful tool to investigate resting brain networks [8], [18]–[27]. This method has the advantages of evaluating the connectivity strength as well as the temporal spatial patterns of interactions on a whole brain scale, by defining a graph as a set of nodes (brain regions) and edges (functional connections) [8], [25], [28]. While a graph represents the functional connection between brain regions, several statistical parameters were used to delineate cortical network hubs and information processing efficiency of the networks under the graph theoretical framework. In this study, we constructed brain networks to characterize the interregional relations between brain regions in CHUs and non-drug users (NDUs) respectively. By applying a cue-reactivity task, we then measured the relationship between drug-related, cues-induced craving activity and abnormal topological properties of CHUs' resting networks.

## Materials and Methods

All research procedures were approved by the Institutional Review Board of the Fourth Military University on Human Studies and were conducted in accordance with the Declaration of Helsinki.

### 2.1 Participants

Sixteen abstinent heroin-dependent males (right-handed, age 36.7±7.1 years, range 25–47 years) were recruited from a local methadone replacement therapy center (three of them were reported in our previous research [6]), and sixteen age-, education- and gender-matched, healthy, right-handed males (age 37.3±6.9 years, range 26–49 years) were recruited from the local community. To confirm the diagnosis of opiate dependence based on the criteria set forth in the DSM-IV, all patients were screened by the Structured Clinical Interview (SCID-IV) for the Statistical Manual of Mental Disorders, Fourth Edition (DSM-IV). Exclusion criteria included psychiatric, neurological, and medical disorders requiring immediate treatment; additional current substance abuse/dependence diagnosis; and contraindications to being scanned. None of the subjects was taking prescription drugs that affected the central nervous system within 1 week of testing. All CHUs had a mean heroin dependence history of 85.3±46.2 months (range 19–182 months); a prior mean daily dosage of 0.6±0.3 g (range 0.2–1.5 g); mean abstinence from heroin for about 4.7±0.7 months (range 3–6 months) and tested negative for morphine in the urinalysis (reagent produced by China Carrie City International Engineering Co.). None of the subjects had a history of neurological illness or injury with the exception of heroin addiction. No patients displayed overt behavioral signs of heroin intoxication upon recruitment. All subjects were fully informed and gave written consent. Information regarding the demographic and clinical information of heroin-dependent individuals and controls is presented in Table 1. The experimental protocol was approved by the Institutional Review Board of the Fourth Military University, China.

**Table 1:** Demographic characteristics of subjects.

Information	Non-drug users (n = 16)	Chronic heroin users (n = 16)
**Age (years)**	37.3±6.9	36.7±7.1
**Education (years)**	8.7±2.1	8.4±1.6
**Duration of heroin use (months)**	N/A	85.3±46.2
**Dosage of heroin use (g/day)**	N/A	0.6±0.3
**Duration of abstinence from heroin(months)**	N/A	4.7±0.7
**Average methadone use (mg/day)**	N/A	34.6±18.1

### 2.2 Experimental paradigm

The present study consisted of a resting state fMRI scan and a drug-related cue-reactivity fMRI experiment. During the resting scan, which was acquired prior to the cue-reactivity task, all participants were instructed to fixate on a visual cross-hair centered on a screen. The resting run lasted 5 min.

During the drug-related cue-reactivity scan, all CHUs were presented with heroin-related and neutral stimuli. Images for heroin-related stimuli contained heroin injection, preparation, and paraphernalia. Neutral images (control) were composed of household objects and tasks. Trials had 2 sec heroin-related or neutral stimuli followed by a variable interval (4–12 sec) during which a crosshair was shown. Images were rear-projected to the center of the visual field via a mirror mounted on the scanner head coil. Stimuli were randomized in an event-related experimental design using E-prime software (Psychology Software Tools, Inc., Pittsburgh). Subjects were placed in the scanner in a supine position using a foam head holder to reduce motion artifacts. Earplugs were used to safely reduce scanner noise. The task was initiated with a 10 sec dummy scan followed by the image. Task duration was 8 min and 10 sec (48 trials). The scan was at least 5–8 hrs after the last methadone dose; a time point when the methadone plasma level was stable with daily dosing [29], [30].

Before and immediately after each imaging session, craving ratings were obtained. Craving was assessed by a 0–10 visual analog scale (VAS), in which participants marked a 0 (“not at all”) to 10 (“extremely high”) in response to the question, “To what extent do you feel the urge to use heroin?”

### 2.3 MR data acquisition

This experiment was carried out in a 3T GE scanner. Prior to the functional run, subjects underwent ‘mock scans’ (gradients 40 mT/m, 150 T/m/sec) in order to become familiar with the scanning environment (for 1 min). A gradient echo T2*-weighted sequence with in-plane resolution of 3.75 mm×3.75 mm (TE 30 ms, TR 2 sec, matrix 64×64, field of view 240 mm, and flip angle 90°, 5 mm slice thickness, no gaps) and a set of T1-weighted high-resolution structural images (TE 3.39 ms, TR 2.7 sec, matrix 256×256, field of view 256 mm, flip angle 7°, in-plane resolution 1 mm×1 mm, and slice thickness 1 mm) were acquired. The resting run generated 150 whole-brain volumes, and 240 whole-brain volumes in each subject were acquired in the drug-related cue-reactivity run.

### 2.4 Data preprocessing

Image preprocessing was carried out using SPM5 (http://www.fil.ion.ucl.ac.uk/spm). All datasets were initially slice time corrected with a reference to the first slice acquired, and then corrected for temporal offsets using sinc interpolation and head movement-related effects using a six-parameter spatial transformation [31]. To minimize movement artifacts, individuals with an estimated maximum displacement in any direction larger than 1 mm or head rotation larger than 1° were discarded from the study. No data were excluded under this criterion. All datasets were then spatially normalized to the Montreal Neurological Institute (MNI) echoplanar imaging (EPI) template image using an optimum 12-parameter affine and nonlinear cosine basis function transformation, and resampled to 3-mm isotropic voxels. In order to avoid local correlations, the spatially normalized data were not spatially smoothed in this study.

### 2.5 Construction of the unweighted voxel-based network

For the resting state dataset, we down-sampled all voxels to 6 mm isotropic and obtained 3446 nodes of interest covering the entire brain to obtain high-resolution brain networks in both the CHU and NDU groups. In order to correct the physiological noise, the fMRI time series of all of the nodes was first filtered using a bandpass filter (0.01–0.08 Hz) to reduce the effects of low-frequency drift and high-frequency noise. Then, the mean time courses from the deep white matter and ventricles were regressed out from the filtered time series. The mean time course from the deep white matter was obtained by averaging the voxel values within a sphere (8 mm radius) positioned in the anterior portion of the right centrum semiovale comprised solely of white matter voxels. The mean time course from the ventricles was obtained by averaging the voxel values within the ventricles using the ventricle mask produced by the WFU PickAtlas Tool [32]. Removal of the global signal would cause a shift in the distribution of the correlation coefficients and make interpretation of the sign of the correlation ambiguous [33]. The global signal was not regressed in the current study. To correct subject motion, we also regressed out the 6 rigid-body motion parameters from the motion correction process from the time series. Finally, a 3446×3446 matrix of the Pearson correlation coefficients was calculated based on the above denoised motion-corrected time courses between all possible connections of the node pairs.

**Figure 1:**
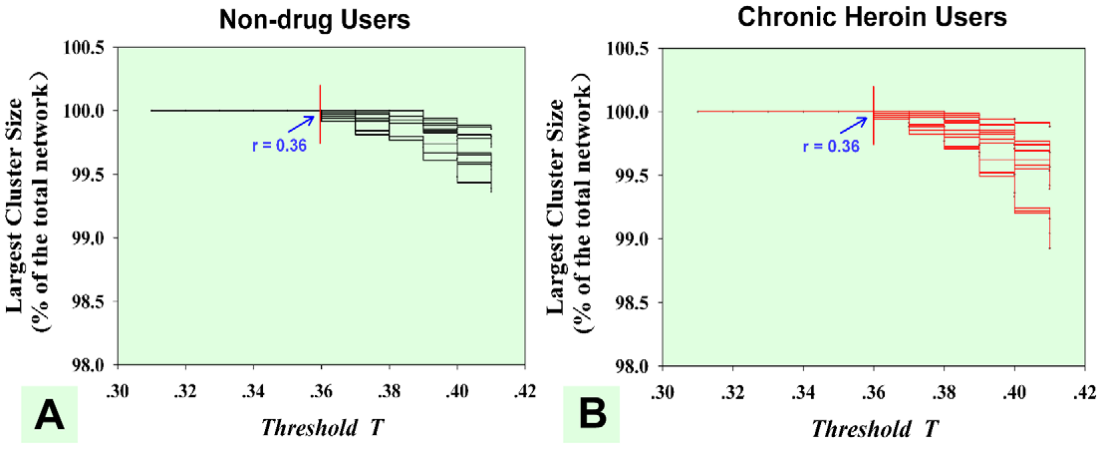
The selection of lower bound threshold T. (A) Largest subgraph size as a function for the NDUs' voxel-based resting networks (black lines) and (B) the CHUs' voxel-based resting networks (red lines). It showed that brain networks were fully accepted in both groups at T
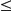
0.36.

We investigated the brain's topological properties by way of binarized graphs (G) in which each correlation matrix was thresholded and converted to the adjacency matrix. The adjacency matrix is a means of representing which nodes of the graph are adjacent to other vertices with 1 being the existing edge and 0 indicating the absence of an edge between the two nodes. A brain functional connection could be represented as an undirected edge if the correlation coefficient between the two nodes achieved a correlation threshold T. Since there is no definitive method in selecting a single threshold, we followed a processing stream that is widely used in threshold selection [34], [35]. We thresholded each correlation matrix repeatedly over a wide range of threshold T from 0.36 to 0.6 in 0.01 increments and then estimated the network properties at each threshold value. We chose R = 0.36 as the lower bound so that each network was fully connected with all of the nodes (n = 3446) (Fig. 1). This allowed the adjacency matrix to be sufficiently sparse for the network metric calculation [27], [34]. The higher bound T = 0.6 was selected to avoid fragmentation of the voxel-based networks. A conservative threshold may destroy the topological architecture of the network and make the following analysis impractical.

### 2.6 Topological properties of the brain functional networks

Small-world models are useful for connectivity studies of nervous systems because they have high clustering and a short path length which confers the capability for both specialized or modular processing in local neighborhoods, as well as including distributed or integrated processing over the entire network [18]. Several network metrics were calculated to assess small-world properties. The key parameters of the small-world network are the clustering coefficient C and the mean minimum path length L. The clustering coefficient 0<Ci<1 is a ratio that defines the proportion of possible connections that actually exist between the nearest neighbors of a node [8], [36]:
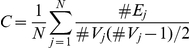
where N is the total number of nodes in the network, 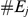 is the number of edges connecting the neighbors of node *j*, and 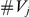 is the number of neighbors of node *j*. The minimum path length 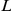 is the average of the shortest path lengths over each possible pair of vertices:
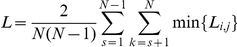
where 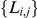 is the shortest path length between the 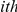 node and the 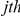 node, and the path length is defined as the number of edges included in the path. Corresponding parameters for a random graph of C and L with the same number of nodes were also calculated, as denoted by 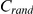 and 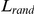. These random networks were generated by randomly reconnecting each edge in the original network an average of 10 times to annihilate any local neighborhood structure while preserving the original degree distribution [34], [37]. Random networks have a small average shortest path length, but with limited local interconnections resulting in a small 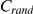 and 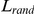. A graph is considered small-world if its average clustering coefficient C is significantly higher than a random graph constructed on the same number of nodes, and if the graph has a small average shortest path length. We examined the ratio 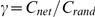 and the ratio 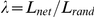 in our voxel-based resting networks. The ratio 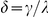 could be summarized for small-world networks as typically being >1 [18]. It has been shown that brain functional networks have economical small-world properties with high global efficiency (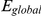) supporting efficient parallel information transfer at a relatively low cost [8], [38]. In GTA, 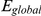 is defined by the inverse of the harmonic mean of the minimum absolute path length between each pair of nodes [38]–[40]:
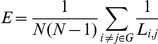



Degree (D) is another important statistical parameter under the graph theoretical framework. The degree of a graph is the number of edges incident to the vertex, and it is defined as the number of nodes across the brain showing a strong correlation with the target node. The brain regions showing a high value of degree in the network were considered to be a cortical hub, which may play a critical role in integrating diverse informational sources and balancing the opposing pressure to evolve segregated, specialized pathways [20].

In our study, we investigated the small-world properties of CHUs' and NDUs' resting networks over the entire range of threshold values, and statistical comparisons of C, L, 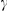, 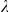, and 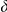 between the two groups were performed by using a two-sample two-tailed *t*-test. For the most significant between-group difference threshold value, the global efficiency of the CHUs' resting network was investigated by examining its association with the duration of heroin use while controlling for age. Since each node has a 3D voxel coordinate in the brain, every node in the voxel-based network could be located in particular anatomical areas [20], [27], [34]. To view all abnormal brain regions in normalized space, the nodes' degree was projected back to the original 3D brain space. Comparing the degree differences between the two groups, we could locate most significant brain regions after a two-sample *t*-test.

### 2.7 Construction of the weighted region-based network

Voxel-based network analyses have the advantage of localizing cortical hubs to particular anatomical areas since each node has a 3D voxel coordinate in the brain [20], [27], [34]. The visualization of a graph helps identify key nodes as well as their topological and spatial relationships with other brain regions [34]. However, since the brain is organized into functional areas, which can be variable in size but are generally larger than voxels in the voxel-based network analysis, the oversampling of functional areas will lead to voxels in larger functional areas that automatically have a functional connection even though there may not be direct functional connectivity. One concern in the voxel-based network analysis is that local spatial correlations may affect the reliability of the network analysis. To exclude potential effects of the local correlation, voxel-based networks were converted into a region-based network to provide a comprehensive assessment of the difference between CHUs' and NDUs' resting networks.

The resting brain is organized into segregated systems of brain areas, and these subsystems interact in a flexible manner to support various cognitive functions [20], [22]. Region-based network analysis could provide excellent network visualization of the functional connectivity between brain regions and identify interacting brain subsystems. Region-based network analyses detected a significant bilateral effect. If the group difference in some brain regions were just subthreshold at one side in the voxel-based network analysis, the differences were regarded as bilateral. In our current study, building upon the degree metrics of CHUs' and NDUs' voxel-based networks, we investigated the distribution of the voxels which showed significant differences for the degree of connectivity for the networks. We then chose the MNI coordinates of the maximally-abnormal voxel within an anatomical area (minimum cluster size 3 voxels) as the center to draw a sphere with a 12 mm diameter. Within each sphere, the voxels located in the white matter and ventricles were removed to ensure the integrity of its structure and function. In this way, we could gain several regions of interest (ROIs). Isolated voxels showing significant regional differences in degree were excluded from the final ROIs. We considered these ROIs as network nodes and created fully connected, weighted, region-based networks for the CHUs' and NDUs' resting datasets. The correlation coefficient between the two regions was preserved as the weight of the edges [35]. In our region-based networks, we used a two-sample two-tailed *t*-test to determine if the intensity of functional connectivity (connection weights) was significantly different between the two groups. Furthermore, we performed partial correlation analyses between the CHUs' resting state functional connectivity intensity and individual duration of heroin use across all subjects while controlling for age.

### 2.8 Cue-reactivity task

The task datasets were filtered using a high pass filter and cut-off at 128 sec. The statistical evaluation was based on a least-square estimation using the general linear model for each run and across each subject. To construct the task model, a set of delta functions for each condition in the experiment was convolved with the hemodynamic response in SPM. For each subject, heroin-related images vs. neutral image contrasts were created and were then entered into a second-level random-effects group analysis.

To test the hypothesis that abnormal resting state activity affected drug-related cues induced activity in the CHUs' brain networks, the degree value of voxels in the CHUs' voxel-based networks exhibiting dysregulated functional connectivity (compared with NDUs') was correlated with the same region's level of BOLD activation during the cue-reactivity task (heroin-related images>neutral images).

## Results

The mean baseline subjective craving score prior to stimulus presentation was 0.6 (±1.4). The mean subjective craving score post-stimulus presentation was 5.5 (±3.2). There were significant differences in CHUs' craving scores before and after the experimental session (*p*<0.001, paired *t*-test).

### 3.1 Abnormal topological properties in CHUs' voxel-based resting network at different correlation thresholds

**Figure 2:**
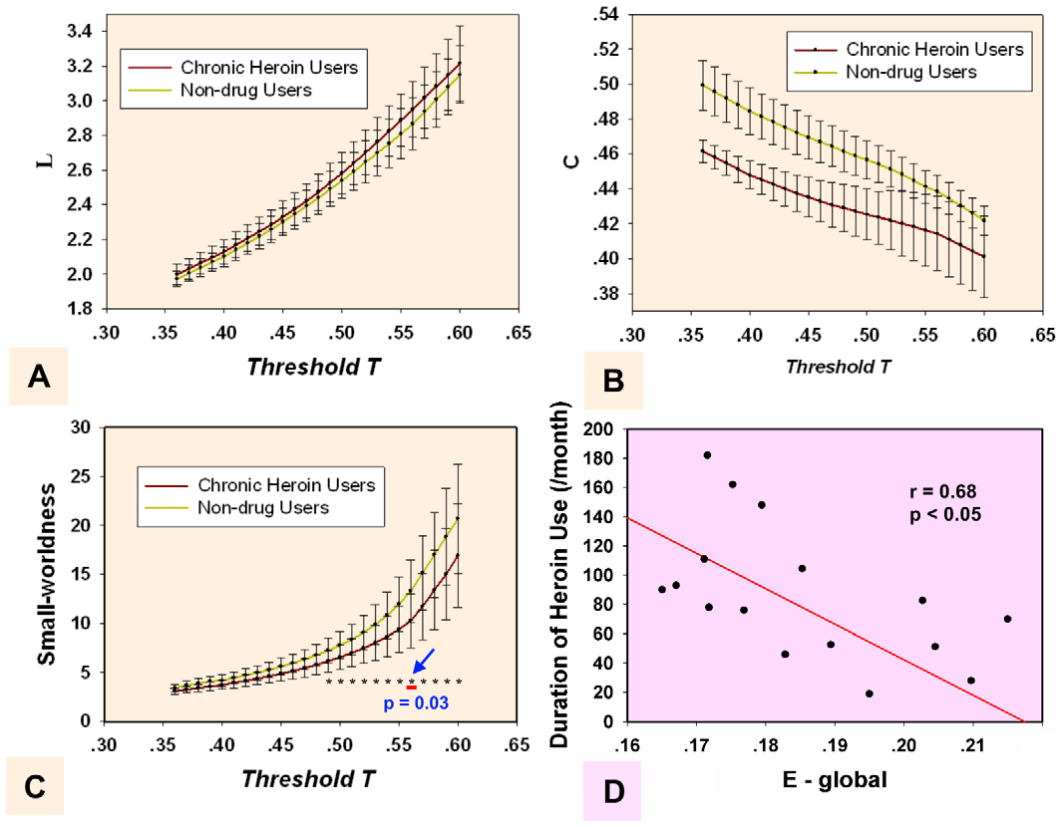
Measures for brain networks topological organization on the whole-brain level. The mean and standard deviation of small-world statistical parameters from voxel-based networks at different correlation coefficient thresholds T. The parameters are: (A) shortest path length L, (B) clustering coefficient C, and (C) small-worldness 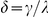. The horizontal dotted line in panel C indicates the significant difference of the small-worldness between the two groups among all of the thresholds. The most significant between-group difference was found at threshold T = 0.56. (D) The correlation between the global efficiency of CHUs' resting networks and the duration of heroin use while controlling for patients' age was at threshold T = 0.56.

Small-world network properties (C, L and 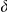) were obtained at different correlation thresholds T (Fig. 2 A, B and C) from 0.36 to 0.6 in 0.01 increments. In our results, the small-worldness ratio 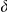 showed a significant difference (*p*<0.05, FDR corrected) in the threshold range between 0.49 and 0.60 for the CHUs' and NDUs' resting networks. The most significant between-group difference was found at threshold T = 0.56 (Fig. 2 C). Yuan et al. (2010) and Liu et al. (2009) previously compared the small-worldness of the brain functional networks between the two groups at a single threshold value and explored abnormal topological properties in the brain of chronic heroin users [7], [8]. Here, we compared the small-worldness between the two groups at a wide range of thresholds and acquired more precise and comprehensive descriptions of the topological properties of the networks.

The results exhibited that global efficiency was negatively correlated with the duration of heroin used by applying a linear partial correlation model controlling for the patients' age when the threshold was below 0.56 in the CHUs' resting networks (*r* = 0.68, *p*<0.05) (Fig. 2 D). Such a correlation analysis between the ability of the information transform (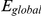) of the network and the duration of heroin use could contribute to revealing brain impairment for prolonged heroin dependence. The network pattern at threshold T = 0.56 for CHUs' and NDUs' networks was shown as being typical in the following analysis.

### 3.2 Dysregulation of brain regions in CHUs' voxel-based resting networks

**Figure 3:**
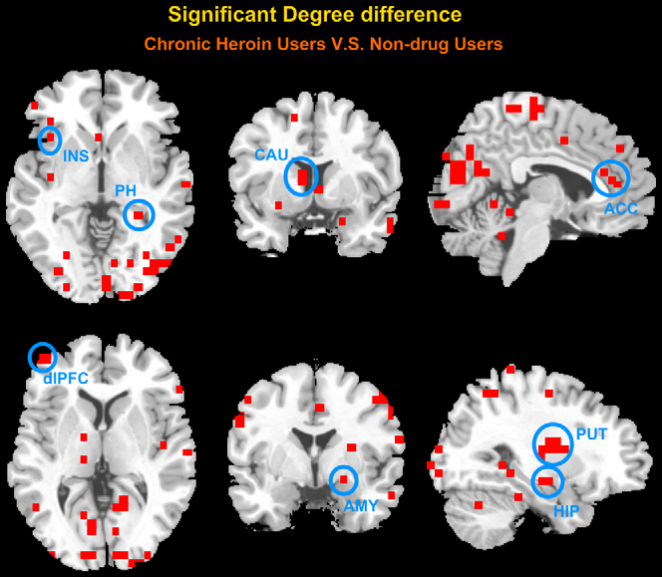
Significant degree differences between CHUs' unweighted voxel-based resting networks and NDUs' resting networks were at a threshold of T = 0.56. A two-sample two-tailed *t*-test was performed (*p*<0.05, corrected). All clusters contain at least three contiguous voxels.

Based on each nodal degree, we performed a two-sample two-tailed *t*-test to determine if the degree of the brain voxels was significantly different between the CHUs' and NDUs' resting networks (p<0.05, corrected). As can be seen from Fig. 3, several brain regions during heroin dependence exhibited functional dysregulation in the resting networks, including the PFC , insula (INS), parahippocampal (PH), thalamus (THA), ACC, posterior cingulate cortex (PCC) , amygdala (AMY), hippocampus (HIP), caudate (CAU), putamen (PUT), pallidus (PAL), temporal cortices, and the anterior/posterior part of the cerebellum (Fig. 3 and Table 2).

**Table 2:** Foci with significant changes in degree differences from non-drug users(NDUs) versus chronic heroin users(CHUs).

Regions of interest		Brodmann	MNI			*NDU Degree*	*CHU Degree*	*p*
		Area	x	y	z	mean ± sd	mean ± sd	value
Inferior Frontal Gyrus (IFG)	L	10/46/47	−15	18	−24	17.4±22.8	80.9±68.5	0.008
	R		39	25	6	42.2±45.4	116.1±88.9	0.03
Medial Frontal Gyrus (medFG)	L	32/9	−3	42	31	47.8±33.4	127±76.8	0.007
	R		3	42	31	41.4±27	103.6±67.9	0.01
Middle Frontal Gyrus (MFG)	L	10/9/6	−45	54	−6	25.1±22.2	176.6±79.3	0.002
	R		27	−12	48	25.5±36.7	133±95	0.004
Superior Frontal Gyrus (SFG)	L	10	−27	60	6	32±36	140.7±131.3	0.012
	R							
Anterior Cingulate Cortex (ACC)	L	24/25	−3	30	12	98.3±57.6	28.3±39	0.004
	R		3	30	12	97.5±43.8	25±30.2	0.0003
Middle Cingulate Cortex (MCC)	L	24/32/31	−15	24	36	2.1±2.4	43±35.2	0.002
	R		21	−18	41	7.8±13.6	58.3±38.7	0.001
Posterior Cingulate Cortex (PCC)	L	23/30/31	−21	−66	6	337.9±297.6	108.9±93.8	0.02
	R		3	−66	12	332.3±299.3	100.8±57.8	0.02
Parahippocampal (PH)	L		−21	−54	0	255.7±188.5	65.5±58.1	0.004
	R		27	−48	−6	273.6±215.3	78±53.7	0.008
Hippocampus (HIP)	L							
	R		27	−12	−19	184.6±170.6	25±26.9	0.006
Amygdala (AMY)	L							
	R		21	0	−18	123±140.7	31.1±29.4	0.048
Insula (INS)	L		−39	−12	12	37.2±33.1	131.2±129.2	0.038
	R		39	−36	18	16.9±26.8	86.9±89.2	0.028
Thalamus (THA)	L		−15	−30	11	47.7±37.4	11.5±19.1	0.01
	R							
Caudate (CAU)	L		−15	18	12	20.3±26.7	93±61	0.002
	R		9	12	12	62.4±29.2	24.7±26.5	0.006
Putamen (PUT)	L		−21	6	6	35.8±16.2	97.7±70.2	0.01
	R		27	−12	12	25±22.8	102.9±61.7	0.001
Pallidus (PAL)	L		−15	−6	−6	39.3±45.3	90±48.2	0.02
	R		21	−6	6	18±15.5	90.5±73.8	0.006
Fusiform (FFG)	L		−51	−42	−24	278.9±181.6	102.3±91.7	0.01
	R		51	−60	−18	336.1±217.1	101.6±86	0.003
Temporal Cortex	L		−63	−36	−18	220.2±157.3	60.6±56	0.005
	R		57	12	−24	235.7±179.5	29±25.2	0.001
Occipital Cortex	L		−39	−96	0	2.7±6	44.9±31.7	0.0005
	R		15	−90	12	290.3±195.1	65.3±48.3	0.001
Cerebellum	L		−9	−48	0	278.1±70.6	83.5±60.3	0.01
	R		9	−48	0	272.8±108.3	89.5±54.1	0.01

### 3.3 Dysfunctional connectivity of brain regions in CHUs' region-based resting networks

**Figure 4:**
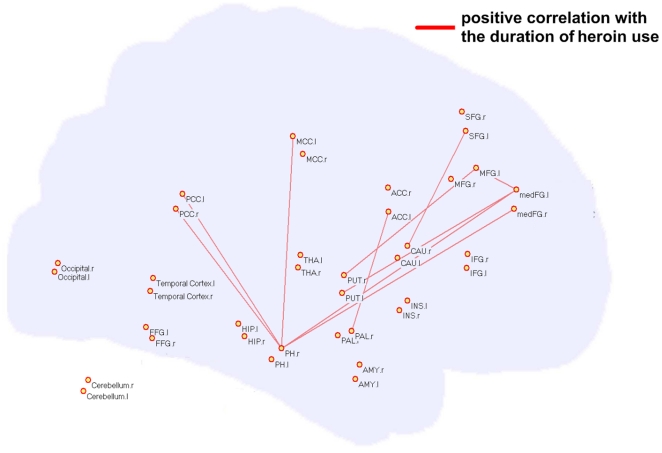
Significant differences in the intensity of the functional connection between the two groups' weighted region-based networks. Ten connections (red lines) showed increased intensity in the CHUs' resting networks (CHUs>NDUs), and a positive correlation with dependence duration in the CHUs' networks while controlling for the patients' age.

**Figure 5:**
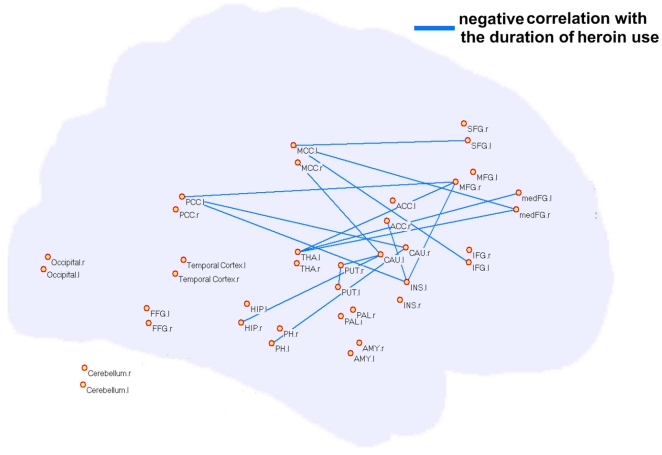
Significant differences in the intensity of the functional connection between the two groups' weighted region-based networks. Sixteen connections (blue lines) exhibited decreased intensity in the CHUs' resting networks (CHUs<NDUs), and a negative correlation with dependence duration in the CHUs' networks while controlling for the patients' age.

While voxel-based network visualization provided strong hints of dysregulated brain regions in the CHUs' networks, local spatial correlations could influence the results of our network analysis. In order to avoid local correlations, we chose abnormal brain regions as the ROIs from the voxel-based network analysis to reconstruct the resting network for a region-based network analysis. Thirty-eight brain regions were considered as network nodes in our analysis. The time course of each ROI was correlated to every other ROI to obtain a 38×38 matrix of correlation coefficients in the CHUs and NDUs groups respectively. A total of 703 connections were obtained in each group. Comparisons of functional connectivity between the two groups' region-based networks were made using a two-sample two-tailed *t*-test. In our results, many pairs of connections were prominently altered (*p*<0.05, corrected). Furthermore, these resting state functional connectivities between brain regions presented a significant correlation with the duration of heroin use. Specifically, ten connections showed a marked difference (CHUs>NDUs) and a positive correlation was present with dependence duration in the CHUs' networks, predominantly involving the PH-PCC, PH-PFC, striatum-PFC, striatum-ACC and a connection within the PFC (red lines in Fig. 4, *p*<0.05). Sixteen connections exhibited decreased intensity in the CHUs' resting networks, including the HIP/PH-striatum, THA-PFC, ACC-INS and a connection within the striatum (blue lines in Fig. 5). These connections showed significant negative correlation with the duration of heroin use (*p*<0.05).

### 3.4 Functional abnormality in cue-reactivity paradigms

**Figure 6:**
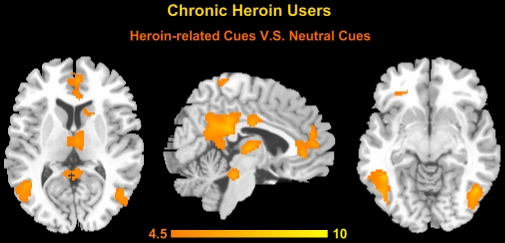
Group results of brain activation contrast for heroin-related images and neutral images (*p*<0.01, FDR corrected, cluster size >20 voxels).

To detect the BOLD signal changes between heroin-related cues and neutral cues, a GLM model was calculated across each subject with regressors for the difference from these two conditions (*p*<0.01, FDR corrected, cluster size >20 voxels as shown in Fig. 6). The heroin-related images and neutral images contrast showed brain activities in the meFG, (BA9, 10), ACC, PCC/precuneus, middle cingulate cortex (MCC), THA, caudate, inferior parietal lobule (IPL), fusiform, occipital cortices, temporal cortices, and the anterior/posterior part of the cerebellum. In contrast, we did not find any area which was more activated for the neutral images than heroin-related images trials.

### 3.5 Nodal degree metrics in the resting network and BOLD activation in task correlations

**Figure 7:**
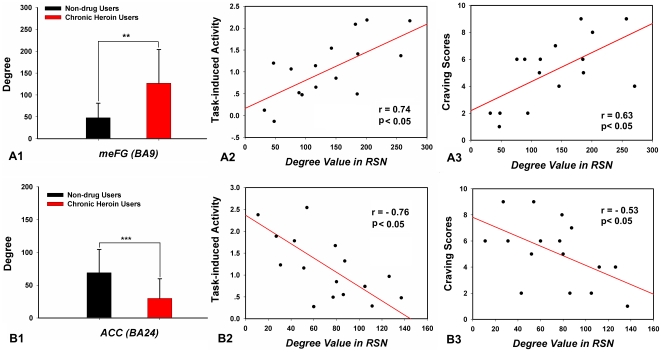
The bars are the degree comparisons between the two groups' resting state networks (RSN) in the medial frontal gyrus (meFG) (A1) and the anterior cingulate cortex (ACC) (B1). Error bars are based on a 95% confidence interval. *p<0.01, **p<0.005. Scatter plots showing the correlation between the heroin-addicted individuals' degree values of the meFG (A2) and the ACC (B2) at rest and the same regions' neural responses induced by a heroin-related cue-reactivity task. Furthermore, the presences of resting deregulations of the meFG and the ACC were relevant to cue-induced craving scores (A3, B3).

In the current study, we only found two regions showing association between resting connectivity and task-related activity (*p*<0.05) in CHUs. The meFG (BA 9, MNI coordinate: −3, 42, 31) showed more functional connectivities (high degree value) at rest in the heroin-addicted group and the degree value of the meFG was positively correlated to the heroin-addicted individual's craving responses in the meFG (r = 0.74, *p*<0.05) (Fig. 7 A2). The degree value of the ACC (BA 24, MNI coordinate: −3, 30, 12) in the patients' resting state network was significantly lower in the CHUs' networks and was negatively correlated with the heroin-related activity in the ACC (r = −0.76, *p*<0.05) (Fig. 7 B2). Furthermore, these resting deregulations of the meFG and the ACC were relevant to cue-induced craving scores (Fig. 7 A3, B3).

## Discussion

Certain studies pointed out that the fundamental function of resting state networks could serve as an indicator for dysregulation in brain connectivity [41], allowing for prediction of task-induced activity in a variety of psychiatric and neurological disorders [13], [14]. Previously, our research group shed light on both local and integrative systems' perspective and detected the CHUs' abnormal resting network characteristics and their associations with the duration of heroin use [5]–[8]. Although different approaches have been used, these studies converge on the observation that the PFC and ACC-related resting networks exhibited abnormal connectivity patterns in the heroin-addicted brain. We inferred that it may lead to the abnormality of the PFC and ACC-related brain functions under a specific task and cause remarkable performances in heroin-dependent individuals. However, this inference needs further investigation.

In the current study, we examined the patterns on how the CHUs' functional connectivity during the resting state interacted with their brain performance on an associative heroin cue-reactivity task. We found a cumulative effect in heroin-addicted individuals that the longer the heroin use, more functional abnormality existed in the resting networks and the greater the craving activity was related to drug cues. Specifically, in patients with heroin addiction, the small-world property and information processing efficiency of the resting networks were prominently disrupted on a whole-brain level (Fig. 2). From a local view, we confirmed that long-term heroin dependence impaired several brain regions' connectivity patterns during the resting state (Fig. 3), and some pivotal interaction among these brain regions showed a significant correlation with the duration of heroin use (Fig. 4 and Fig. 5). Furthermore, stronger dysfunctional connectivity patterns at rest were associated with greater drug-related cues-induced activities (Fig. 7).

The major finding of our study is that the addicted individual differences in dysregulated functional connectivity of the PFC (meFG, BA9) and ACC at rest correlated with individual differences in the magnitude of the same region's drug-related craving activities and patients' self-report craving scores (Fig. 7). The PFC was largely considered to be involved in cognitive control of goal-directed behavior and rewarded tasks [42]–[45], and referred to the management of integrating cognitive and motivational information [45]. For drug addiction studies, several researchers observed dysfunctional activity of the PFC in decision-making tasks [43], which need heroin dependence patients to integrate cognitive and motivationally relevant information [45]. In our results, patients presenting more abnormal functional connections in the meFG during the resting state tended to have greater neural activity in the meFG for heroin-related cues (Fig. 7). Therefore, our results indicated that the decision-making network is involved in processing an increase in the craving response for heroin-related cues in CHUs. Similarly, our results showed that less functional connections of the ACC during the resting state were associated with greater neural activity between heroin-related cues and neutral cues (Fig. 7). Previous studies have disclosed deactivation of the ACC in a GO/NOGO task in CHUs [2], [46], suggesting the ACC's important role in response inhibition and inhibitory control behavior [3], [6], [47], [48]. The irregularity of the ACC may lead the inhibitory control to become weaker in CHUs [8], therefore, the negative correlation of the ACC between rest and the task may reveal the role of the inhibitory network for heroin-related cue processing.

Heroin addiction was characterized by uninhibited behavior and loss of self-control for drug seeking, which far exceeded the desire for other non-drug-related goals [49]. It suggested that long-term heroin use aggravated drug craving and arousal, and “wanting” of the drug turned these brain functions of addicted individuals into an ill-motivated state [1]. In our results, when considering the rest-task interactions, functional dissociations were apparent during the heroin-related craving task. Functional connection of the meFG and ACC showed different correlation patterns with activity to heroin cues in CHUs (Fig. 7). Our findings provided direct evidence for making inferences about the presence of multiple brain circuits in enhancing salience of the cues to heroin dependent patients. We postulated that the abnormal interaction of these brain regions may be indicative of a trend of the deterioration of monitoring and inhibitory controls for the long-term duration of heroin dependence, thus possibly leading to maladaptive craving responses in heroin dependent patients' daily lives [1], [8], [17].

While dysregulation of the meFG and ACC in the resting network was related to behavioral variability within and across heroin-dependent individuals, several brain regions concomitantly showed dysregulated functional connectivity in CHUs' resting networks, but had no significant correlation with the same brain regions' craving activities (*p*>0.05). In the current study, abnormal topological properties were explored in the major parts of the striatum (CAU, PUT, and PAL), PH, HIP and PCC/precuneus (Fig. 4 and Fig. 5). While connections between the striatum and PFC and the PH and PFC were reinforced with long-term heroin use (Fig. 4), the functional links were weakened within the striatum and its connection to the PH and HIP (Fig. 5). Some animal studies demonstrated that intermittent footshock stress could induce heroin seeking in rats after prolonged drug-free periods [50]. Pruessner et al. (2004) pointed out that increased dopamine release in the striatum was associated with stressful experiences and reward processing [51]. The PH and HIP were considered to be involved in the mesolimbic dopamine circuit and were referred to as being the memory response for drug reinforcement [49]. These abnormal functional organizations in our results may indicate maladjusted, stress-related brain activation and memory-related activities during the dependent state. However, we failed to assess the relationship between these regions' resting connections and drug-related cues-induced activities in CHUs. Further evidence needs to be investigated using a more integrated and comprehensive task design to study behavioral and neuropsychological deficits in heroin-dependent individuals.

There is a methodological limitation to the present study. In our voxel-based analysis, the most significant between-group small-worldness difference was chosen as a typical one for ROI selection. It is plausible that any single threshold T may influence the topological properties of the resting network, which may yield different results of ROI selection for the region-based network analysis. In our analysis, results of degree comparisons were similar for other threshold values (0.49 to 0.6). Although the number of voxels within an anatomic brain region changed quantitatively for different thresholds, no additional brain regions were found. The sample size used was fairly minute and all abstinent heroin-dependents were male, which may have resulted in a non-significant correlation between the network parameter and the performance mentioned previously. A larger sample size is pertinent in future studies to be statistically significant in validating these results.

In summary, drug addiction is characterized as a complex brain disease [1], [49]. In this study, we were able to combine the analysis of functional connectivity measures obtained at rest and during addiction-induced brain performance for a heroin-related craving task. Our results may suggest that there exist two brain systems interacting simultaneously in the heroin-addicted brain for heroin-related cues. These findings may help us better understand the issues of heroin-related behavioral and addicted mental states. Further research is needed to apply integrated and comprehensive task design to determine whether or not impaired functional connectivity between two brain regions will serve as a predictor to heroin-related brain activities.
